# Itaconate negatively regulates innate immunity during fungal pneumonia

**DOI:** 10.1128/mbio.01491-26

**Published:** 2026-07-27

**Authors:** Thomas Draper, Pari Dhruva, MaryJane Jones, Rachel McCarley, Christine M. Bojanowski, Chad Steele

**Affiliations:** 1 Department of Microbiology and Immunology, School of Medicine, Tulane University 5783, New Orleans, Louisiana, USA; 2 Department of Medicine, Section of Pulmonary Diseases, Critical Care and Environmental Medicine, School of Medicine, Tulane University 5783, New Orleans, Louisiana, USA; Instituto Carlos Chagas, Curitiba, Brazil

**Keywords:** innate immunity, fungal, lung defense

## Abstract

The innate immune response to pathogens often involves metabolic reprogramming, such as disruption of the Krebs cycle, leading to the accumulation of various metabolites that can further influence innate cell responses during infection. Of these, the immunomodulatory metabolite itaconate has been shown to positively or negatively influence lung immune responses, depending on the pathogen. In the current study, we found that mice deficient in aconitate decarboxylase (*Acod1*−/− mice), the enzyme that produces itaconate from cis-aconitate, cleared the opportunistic mold *Aspergillus fumigatus* from the lung more effectively than wild-type (WT) control mice. Augmented fungal clearance in *Acod1*−/− mice correlated with increased type 17 responses, which themselves correlated with higher IL-1β, PGE2, and γδ T cell levels. Intriguingly, we show that alveolar macrophages and neutrophils from naïve *Acod1*−/− mice kill *A. fumigatus* more efficiently. Conversely, the addition of exogenous itaconate to alveolar macrophages and neutrophils from naïve WT mice reduced their antifungal capacity. Mechanistically, alveolar macrophages, but not neutrophils, from naïve *Acod1*−/− mice demonstrated enhanced ROS production when stimulated with *A. fumigatus*. We further show that mice with macrophage-specific, but not neutrophil-specific, *Acod1* deficiency cleared *A. fumigatus* more effectively. Itaconate deficiency also protected against fungus-induced mortality during corticosteroid-mediated immunosuppression. Finally, itaconate reduced the antifungal activity of human monocyte-derived macrophages. Collectively, these data identify an immune regulatory role for itaconate during *A. fumigatus* fungal pneumonia and potentially identify a new therapeutic target for enhancing protection against *A. fumigatus*.

**IMPORTANCE:**

Fungal infections by invasive molds such as *Aspergillus fumigatus* are leading causes of morbidity and mortality in immunocompromised individuals, such as patients with hematologic malignancies, and recipients of hematopoietic stem cell transplant (HCT), solid organ transplant (SOT), and cellular therapies. A major shift in the increased incidence of these infections is a result of a rapidly expanding global immunocompromised population due to targeted immunotherapies and biologics for the treatment of cancer, combination therapies, cellular therapies, and bispecific and trispecific antibody therapies. The advancement in these immunomodulatory/immunosuppressive therapies is outpacing our understanding of mechanisms that lead to the development of infections such as invasive aspergillosis. Therefore, the continuing evolution of our understanding of protective and immunoregulatory responses would be expected to reveal new mechanisms that govern susceptibility to fungal pneumonia. To this end, in the current report, we show that the TCA cycle intermediate itaconate hinders lung clearance of *A. fumigatus* via regulating multiple immune mechanisms. Overall, our study uncovers a new mechanism of immune regulation during fungal pneumonia.

## INTRODUCTION

Severe fungal infections have emerged as a significant global health concern over the past decade, with over 6 million infections worldwide causing more than 2.5 million deaths annually (1). With respect to invasive pulmonary aspergillosis (IPA), it is estimated that more than 2 million people develop disease in the context of chronic obstructive pulmonary disease, intensive care, lung cancer, or hematological malignancy, with a crude annual mortality of nearly 85% (1.8 million). This alarming increase is thought to be a result of increased usage of immunosuppressive therapies, growing resistance to antifungal drugs, and even global warming (1, 2). In October 2022, the World Health Organization published the first-ever fungal priority pathogens list (FPPL), which examined nineteen pathogens, ranking four of them as of critical importance, which included the opportunistic mold *Aspergillus fumigatus*. Invasive fungal pneumonia such as invasive aspergillosis is considered to be a result of profound immunosuppression, particularly with prolonged neutropenia and lymphopenia. However, it is becoming more apparent that IPA can affect a much broader population, including patients with more subtle Immunosuppression (2). Indeed, corticosteroid treatment is a major risk factor for developing IPA (3). This has been reinforced by the observation of increased incidence of IPA in virally-infected patients, which correlated with steroid usage (4–6). Collectively, there is an ongoing need to better understand how modulation of the immune response affects susceptibility to fungal infections in order to discover new mechanisms of harnessing the immune response to protect against infection.

An infectious process often results in metabolic stress in both immune cells and tissues, with the invading pathogen simultaneously attempting to use this stress to its advantage. Indeed, this has been studied in lung infection with *Aspergillus fumigatus*, whereby infection often results in a hypoxic environment which facilitates organism adaptation (7, 8). An element of this adaptation for *A. fumigatus* includes thickening of the fungal cell wall, which may give the pathogen an advantage by increasing antifungal drug resistance. However, cell wall thickening also increases the display of PAMPs such as beta-glucans that result in higher inflammatory responsiveness (9). In some cases, however, the inability of immune cells to handle hypoxic environments, loss or downregulation of the mammalian transcription factor hypoxia inducible factor 1α (HIF1α), for example, results in increased innate cell apoptosis and susceptibility to infection *with A. fumigatus* (10). Hypoxia is also known to induce mitochondrial stress (11). A pathway that is aberrantly affected during mitochondrial stress is the energy-generating tricarboxylic acid (TCA) cycle (a.k.a. the Kreb’s cycle). During cellular stress, mitochondria are disrupted, causing both TCA cycle metabolites and their intermediates to be released into the cytosol where they can signal (12). Several of these TCA cycle intermediates, such as itaconate, have garnered attention as immunomodulatory factors, particularly in innate cells such as macrophages and neutrophils. Itaconate is derived from the enzymatic conversion of cis-aconitate by aconitate decarboxylase 1 (ACOD1) (also known as immunoregulatory gene 1, IRG1). Itaconate interrupts the TCA cycle in mitochondria at the enzymatic level of succinate dehydrogenase and has anti-inflammatory and anti-oxidant properties via induction of KEAP1 protein alkylation, which boosts Nrf2 and glutathione levels in myeloid cells, promoting an anti-inflammatory phenotype (13). In terms of lung infection, itaconate has been shown to positively or negatively regulate immune responses during bacterial pneumonia, depending on the pathogen (14–16). In the current report, we describe a role for itaconate in regulating inflammatory responses, including type 1 and type 17 cytokine responses as well as macrophage antifungal activity, which are required for survival and fungal clearance during experimental invasive aspergillosis.

## MATERIALS AND METHODS

### Mice

Male and female age-matched C57BL/6NJ mice, 6 to 8 weeks of age, were obtained from The Jackson Laboratory (Stock #005304). Male and female age-matched *Acod1*−/− mice were obtained from The Jackson Laboratory (C57BL/6NJ-*Acod1^em1(IMPC)J^*/J, Stock #029340) and bred at Tulane University. *Acod1*^fl/fl^ mice (kindly provided by Dr. Sharmila Nair, University of Louisville) (17) were bred with B6.Cg-Tg(Itgax-cre)1-1Reiz/J (Cd11c Cre; Jackson Labs Stock #008068). All animals were housed in a specific pathogen-free facility.

### Preparation of *A. fumigatus*; *in vivo* challenge; lung fungal burden assessment

*A. fumigatus* isolate 13073 (ATCC, Manassas, VA) was maintained on potato dextrose agar for 5–7 days at 37°C. Conidia were harvested by washing the culture flask with 50 mL of sterile phosphate-buffered saline supplemented with 0.1% Tween 20. The conidia were then passed through a sterile 40 μm nylon membrane to remove hyphal fragments and enumerated on a hemocytometer. For *in vivo* challenge, mice were lightly anesthetized with isoflurane and administered 7 × 10^7^ *A. fumigatus* conidia in a volume of 50 μL intratracheally as we have previously reported (18–20). For lung fungal burden analysis, the left lungs were collected at 24 h post-exposure and homogenized in 1 mL of PBS. Total RNA was extracted from 0.1 mL of unclarified lung homogenate using the MasterPure yeast RNA purification kit (Epicentre Biotechnologies, Madison, WI), which includes a DNase treatment step to eliminate genomic DNA as we have previously reported (18–21). Total RNA was also extracted from serial 1:10 dilutions of live *A. fumigatus* conidia (10^1^–10^9^) and DNase treated to form a standard curve. Lung *A. fumigatus* burden was analyzed with real-time PCR measurement of the *A. fumigatus* 18S rRNA (22) and quantified using a standard curve of *A. fumigatus* conidia as we have extensively reported (18–21).

### Lung cell isolation and culture and inflammatory mediator analysis

For lung cell isolation, the lungs were collected and minced in IMDM media (MilliporeSigma) supplemented with 1% penicillin-streptomycin-glutamine (Mediatech), 10% heat-inactivated FBS (Invitrogen), and 0.4 mg/ml polymyxin B (Thermo Fisher Scientific), followed by incubation for 60 min with tissue culture grade type IV collagenase (1 mg/mL; MilliporeSigma) in a 37°C orbital shaker at 100 rpm. The cell suspension was filtered through sterile 70 μm and 40 μm nylon filters, and red blood cells were lysed with ACK buffer (Lonza) to create single-cell preparations. One million cells in a volume of 200 μL were cultured overnight with 1 million *A. fumigatus* conidia (1:1), followed by collection and clarification of supernatants. Supernatants were analyzed for protein levels of 32 cytokines and chemokines using a Milliplex multiplex suspension cytokine array (Millipore) according to the manufacturer’s instructions. The data were analyzed using Bio-Plex Manager software (Bio-Rad Laboratories). PGE2 and IL-22 levels were quantified by ELISA (R&D Systems).

### Flow cytometry

Lung cells were isolated as described above. Cells were washed and Fc receptors were blocked with Mouse BD Fc Block (BD Biosciences, San Diego, CA) at 4°C for 20 min. Thereafter, cells were stained with a single-color LIVE/ DEAD Fixable Dead Cell Stain (Invitrogen) followed by labeling with specific immune cell surface markers. The following staining parameters were employed: eosinophils as CD45+, CD11b+ Siglec F+ Ly-6G−, neutrophils as CD45+, CD11b + Ly6G+, inflammatory monocytes as CD45+, CD11b+ Ly6C + CCR2+, alveolar macrophages as CD45+, CD11c+, CD11b−, F4/80+, Ly6C−, interstitial macrophages as CD45+, CD11c−, CD11b+, F4/80+, dendritic cells as CD45+, CD11c+, Ly6C+, MHC II+, monocyte-derived dendritic cells as CD45+, CD11C+, IA IE+, CD26−, CD88+, Sirpa+, Xcr1−, CD64+, FceR1a+, γδ T cells as γδ TCR+, CD3+ and invariant NKT cells as PBS57-labeled CD1d tetramer+ (all antibodies purchased from Biolegend, eBiosciences, and BD Biosciences).

### Assessment of *in vitro A. fumigatus* killing by alveolar macrophages and neutrophils

Bronchoalveolar lavage was performed on C57BL/6NJ and *Acod1*−/− mice to isolate alveolar macrophages as previously described (18). For *A. fumigatus* killing *in vitro*, macrophages (1 × 10^5^) were co-cultured at a 1:1 effector to target ratio with live *A. fumigatus* resting conidia for 24 h followed by RNA isolation with the MasterPure yeast RNA purification kit and real-time PCR assessment as described above. Controls included *A. fumigatus* resting conidia cultured in the absence of macrophages for 24 h. Bone marrow cells were collected from the femurs and tibiae of C57BL/6NJ and *Acod1*−/− mice by flushing the opened bones with Iscove’s modified Dulbecco’s medium (IMDM; Invitrogen). Neutrophils were isolated from bone marrow cell pellet using Neutrophil isolation kit (Miltenyi). For *A. fumigatus* killing *in vitro*, neutrophils (1 × 10^6^) were cocultured at a 1:1 E:T ratio with live *A. fumigatus* swollen conidia for 24 h, followed by RNA isolation with the MasterPure Yeast RNA Purification Kit and real-time PCR assessment as described above. Controls included *A. fumigatus* swollen conidia cultured in the absence of neutrophils for 24 h. In specific experiments, AMs and neutrophils were cultured in the presence of itaconate (10 mM, pH-corrected, Cat. #I29204, MilliporeSigma) and *A. fumigatus* at a 1:1 E:T ratio (23, 24). Itaconate was confirmed to have no effect on cell viability as assessed via trypan blue exclusion (data not shown). *A. fumigatus* resting conidia cultured with vehicle or itaconate served as controls for viability. After 24 h, the total contents of each well were collected, and fungal killing was assessed by real-time PCR analysis of *A. fumigatus* 18S rRNA levels.

### Assessment of reactive oxygen species

Alveolar macrophages and neutrophils were isolated as described and cultured 1:1 with *A. fumigatus* resting conidia at 37°C in the presence of CellROX Green (5 µM; Invitrogen/Thermo Fisher, Catalog #C10444). Reactive oxygen species levels were measured using a fluorescent plate reader (485 nm) every 30 min for 2 h.

### Corticosteroid-mediated immunosuppression

WT and *Acod1−*/− mice were administrated triamcinolone acetonide (Kenalog, 40 mg/kg intraperitoneally) 24 h prior to *A. fumigatus* challenge. Thereafter, mice were lightly anesthetized with isoflurane and administered 7 × 10^6^ *A. fumigatus* conidia in a volume of 50 μL intratracheally, and survival was followed for 4 days. In specific experiments, fungal burden was assessed at 48 h after challenge as described above.

### Human monocyte-derived macrophages

Peripheral blood samples (10 mL) from healthy human volunteers were drawn into BD Vacutainer Blood Collection Tubes (EDTA K2) and peripheral blood mononuclear cells isolated via Ficoll-Paque PLUS (MilliporeSigma, Catalog #GE17-1440-02). Monocytes were isolated using a human Pan Monocyte Isolation Kit (Miltenyi Biotec Catalog #130-096-537) and cultured with human recombinant M-CSF (50 ng/mL; STEMCELL Technologies Catalog #78057) and ImmunoCult-SF Macrophage Medium (STEMCELL Technologies, Catalog #10961) for 4 days. Monocyte-derived macrophages (MDMs) (1 × 10^5^) were co-cultured at a 1:1 effector to target ratio with live *A. fumigatus* resting conidia in the presence or absence of vehicle or itaconate (10 mM) for 24 h followed by RNA isolation with the MasterPure yeast RNA purification kit and real-time PCR assessment as described above. Controls included *A. fumigatus* resting conidia cultured in the absence of macrophages for 24 h.

### Histology

The left lungs were collected and fixed in 4% formalin. The fixed lungs were paraffin embedded and then processed and stained by GNO Histology Consultants (New Orleans, LA). Imaging was performed using a Swift Optical Instruments M10T-P Trinocular LED Microscope equipped with a Motic Moticam 5 + 5 megapixel digital camera. In specific experiments, *A. fumigatus* organisms were quantified in 40 random high-power fields (40×) from multiple WT and *Acod1*−/− mice.

### Statistics

Data were analyzed using GraphPad Prism Version 10.3.1 statistical software. Comparisons between groups when data were normally distributed were made with the Student’s *t*-test. Significance was accepted at a value of *P* < 0.05.

## RESULTS

### Itaconate deficiency results in enhanced fungal clearance during *Aspergillus fumigatus* lung infection

**Fig 1:**
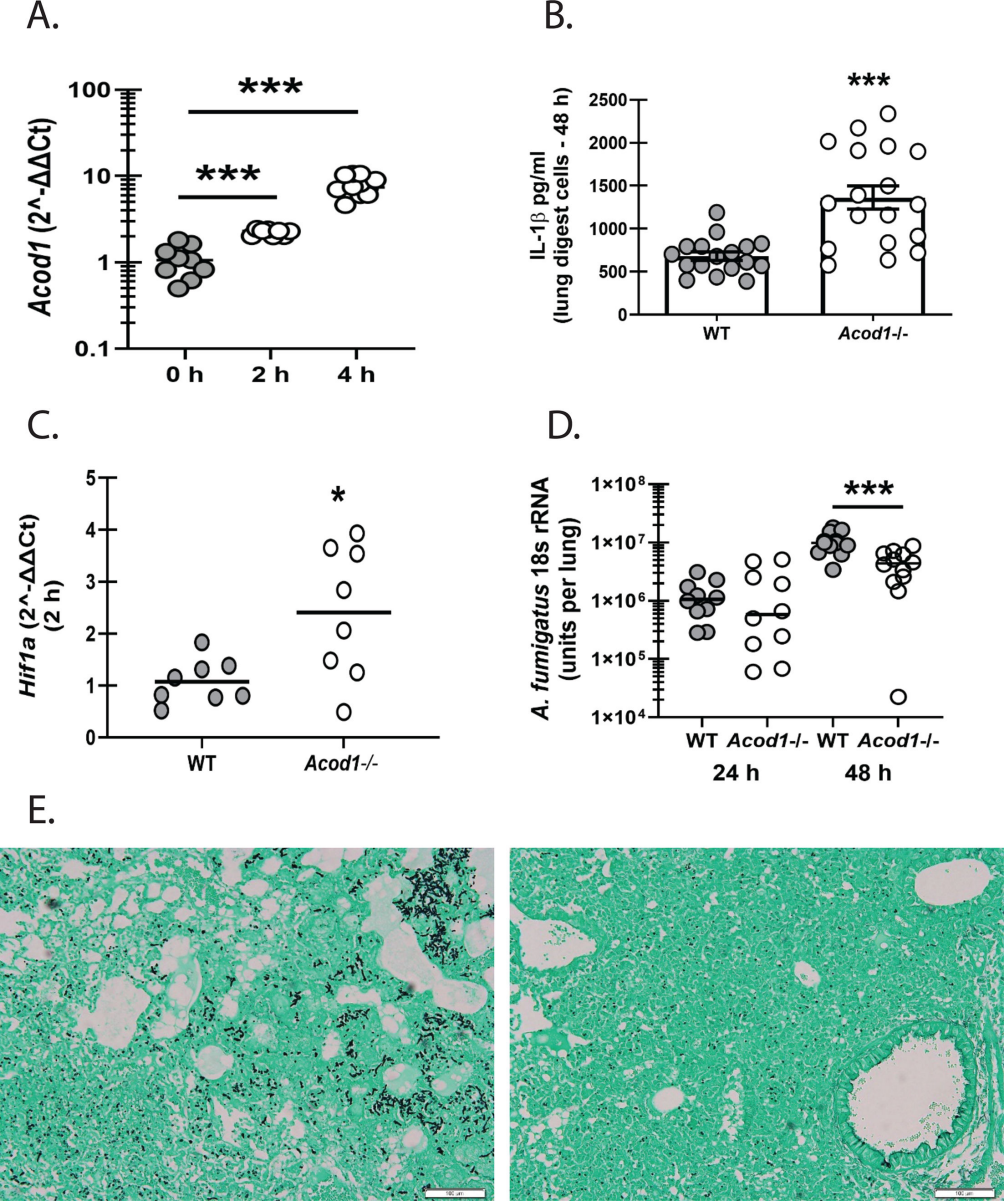
Itaconate deficiency results in enhanced fungal clearance during *Aspergillus fumigatus* lung infection. (**A**) Alveolar macrophages were isolated by bronchoalveolar lavage and cultured in the presence or absence of *A. fumigatus* resting conidia at a 1:1 E:T ratio for 2 and 4 h. *Acod1* gene expression was quantified by real-time PCR and normalized to HPRT. Data are expressed as 2−ΔΔCt. The panel illustrates cumulative data from two independent studies (*n* = 4 or 5 mice per time point per study). (**B**) C57BL/6NJ wild-type (WT) and itaconate-deficient (*Acod1*−/−) mice were challenged intratracheally with 7 × 10^7^ *A. fumigatus* conidia. At 48 h post-challenge, lungs were collected, enzymatically digested, and cultured for 24 h. IL-1β levels were quantified in co-culture supernatants by Luminex-based Milliplex assessment. The panel illustrates cumulative data from eight independent studies (*n* = 5 or 6 mice per group, per study). (**C**) Alveolar macrophages were isolated from WT and *Acod1*−/− mice and cultured as in panel **A**. *Hif1a* gene expression was quantified by real-time PCR and normalized to HPRT. Data are expressed as 2−ΔΔCt. The panel illustrates cumulative data from two independent studies (*n* = 4 mice per time point per study). (**D**) Mice were infected as in panel **A**. Lung fungal burden at 24 and 48 h post-challenge was assessed by real-time PCR analysis of *A. fumigatus* 18S rRNA levels. The panel illustrates cumulative data from two independent studies (*n* = 5 or 6 mice per group per study). For all graphs, each symbol represents an individual mouse, the line within a given group represents the mean, and * and *** represent a *P* value of <0.05 and <0.001, respectively (unpaired two-tailed Student’s *t*-test). (**E**) Representative GMS-stained lung sections from WT mice (left) and *Acod1*−/− mice (right). Original magnification 40×. Scale bar equals 100 μm.

Itaconate promotes an anti-inflammatory response in myeloid cells (25), suggesting that in its absence, heightened inflammatory responses could damage the lung and compromise clearance. Indeed, the absence of itaconate is deleterious during lung infection with *Mycobacterium tuberculosis* (17) or *Klebsiella pneumonia* (15), both as a result of inflammation-mediated pathology. Likewise, inflammatory responses, such as IL-1β signaling, may be detrimental for lung clearance of *A. fumigatus* (26, 27). As itaconate is highly expressed in innate immune cells (28), we first questioned whether alveolar macrophages exposed to *A. fumigatus* expressed aconitate decarboxylase 1 (*Acod1*). Result shows that within 2 h of culture, there was a twofold increase in *Acod1* mRNA levels, which further increased by more than sevenfold at 4 h (Fig. 1A). We next show that the absence of itaconate (*Acod1*−/− mice) results in elevated IL-1β levels in the lung during *A. fumigatus* infection (Fig. 1B), suggesting the possibility that the absence of itaconate may be detrimental for lung fungal clearance. Moreover, hypoxia inducible factor 1α (*Hif1a*), which is critical for *A. fumigatus* lung defense (10) and promotes IL-1β induction (29), was increased in AMs from *Acod1*−/− mice (Fig. 1C). Despite enhanced *Hif1a* and IL-1β induction, itaconate deficiency resulted in a ~60% reduction in lung fungal burden by 48 h post-challenge (Fig. 1D). Grocott-Gomori’s methenamine silver (GMS) staining of lung tissues also indicated higher numbers of *A. fumigatus* organisms in WT mice (Fig. 1E, left) compared to *Acod1*−/− mice (Fig. 1E, right). These data suggest that enhanced inflammation in the absence of itaconate promotes more effective clearance of *A. fumigatus*.

### Itaconate deficiency augments type 17 responses during *Aspergillus fumigatus* lung infection

**Fig 2:**
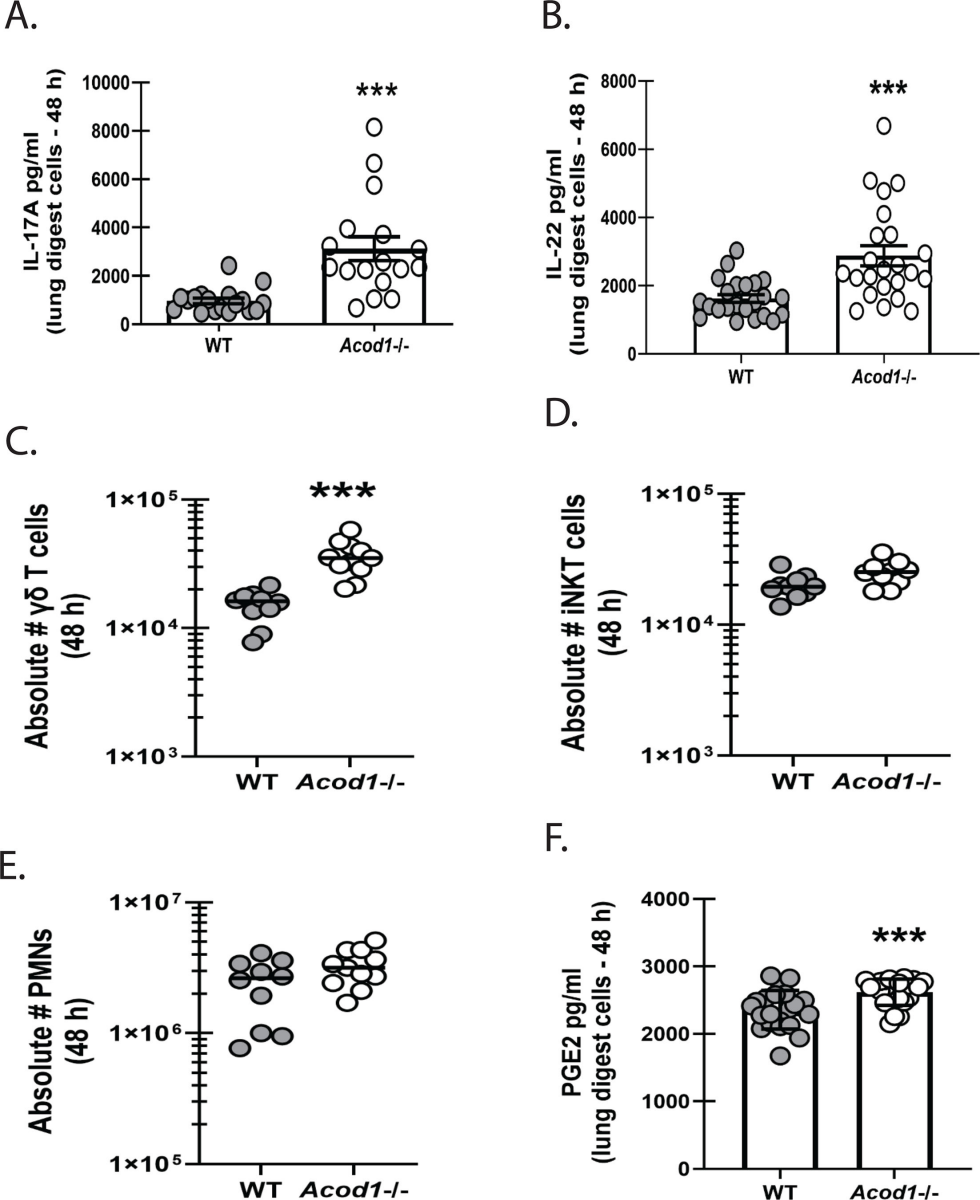
Itaconate deficiency augments type 17 responses during *Aspergillus fumigatus* lung infection. C57BL/6NJ wild-type (WT) and itaconate-deficient (*Acod1*−/−) mice were challenged intratracheally with 7 × 10^7^ *A. fumigatus* conidia. At 48 h post-challenge, lungs were collected, enzymatically digested, and cultured for 24 h. (**A**) IL-17A and (**B**) IL-22 levels were quantified in co-culture supernatants by Luminex-based Milliplex assessment (**A**) or ELISA (**B**). The panels illustrate cumulative data from five to eight independent studies (*n* = 5 or 6 mice per group, per time point, per study). (**C–E**) Lung digest cells were isolated as in panel **A**. (**C**) γδ T cells, (**D**) iNKT cells, and (**E**) neutrophils (PMNs) were quantified by flow cytometry. The panels illustrate cumulative data from two independent studies (*n* = 4 or 5 mice per group, per time point, per study). (**F**) PGE2 levels were quantified in lung digest cell supernatants by an ELISA. The panel illustrates cumulative data from six or seven independent studies (*n* = 5 or 6 mice per group, per time point, per study). For all graphs, each symbol represents an individual mouse, the line within a given group represents the mean and *** represents a *P* value of <0.001 (unpaired two-tailed Student’s *t*-test).

We (19, 20, 30, 31) have shown that type 17 responses (IL-17A and IL-22) are essential for the protection against lung infection with *A. fumigatus*. We have reported that IL-17A is produced by neutrophils during *A. fumigatus* lung infection (30), whereas iNKT cells and γδ T cells are the primary sources of IL-22 during IA (19) (and likely serve as lung cell sources of IL-17A as well). We have shown that the type 17 response peaks in the lung 48 h after *A. fumigatus* challenge (19). To determine if these responses are modulated by the absence of itaconate, we analyzed IL-17A and IL-22 production by lung cells from WT and *Acod1*−/− mice. Results demonstrated that IL-17A (Fig. 2A) and IL-22 (Fig. 2B) were negatively affected by itaconate, in that their levels were significantly higher in infected *Acod1*−/− mice. Other mediators that were also observed to be higher in *Acod1*−/− mice included IL-5 (WT 783 ± 60 vs *Acod1*−/− 1,590 ± 127 pg/mL, *P* = 0.0001) and CCL5 (WT 200 ± 13 vs *Acod1*−/− 290 ± 14 pg/mL, *P* = 0.0001). Itaconate is known to regulate inflammation via the induction of IL-10 (32, 33); however, we did not observe differences in IL-10 between WT and *Acod1*−/− mice (WT 1,054 ± 93 vs *Acod1*−/− 1,127 ± 113 pg/mL, *P* = 0.619). To further investigate mechanisms associated with higher levels of IL-17A and IL-22, we examined the presence of innate-like lymphocytes in the lung, which demonstrated increased numbers of γδ T cells in *Acod1*−/− mice (Fig. 2C), but no differences in iNKT cells (Fig. 2D). We further observed no differences in the levels of neutrophils in the lung (Fig. 2E). We have previously shown that type 17 responses are highly dependent on IL-1R signaling (20). We have further reported that PGE2 was partially dependent on IL-1R signaling and that the inhibition of PGE2 impaired type 17 responses (20). Data in Fig. 1B demonstrated that IL-1β levels were increased in *Acod1*−/− mice, which correlated with increased levels of PGE2 in *Acod1*−/− mice (Fig. 2F). These data suggest that enhanced fungal clearance in the absence of itaconate is augmented by IL-1β and PGE2-driven type 17 responses.

### The absence of itaconate results in increased type 1 responses during *Aspergillus fumigatus* lung infection

**Fig 3:**
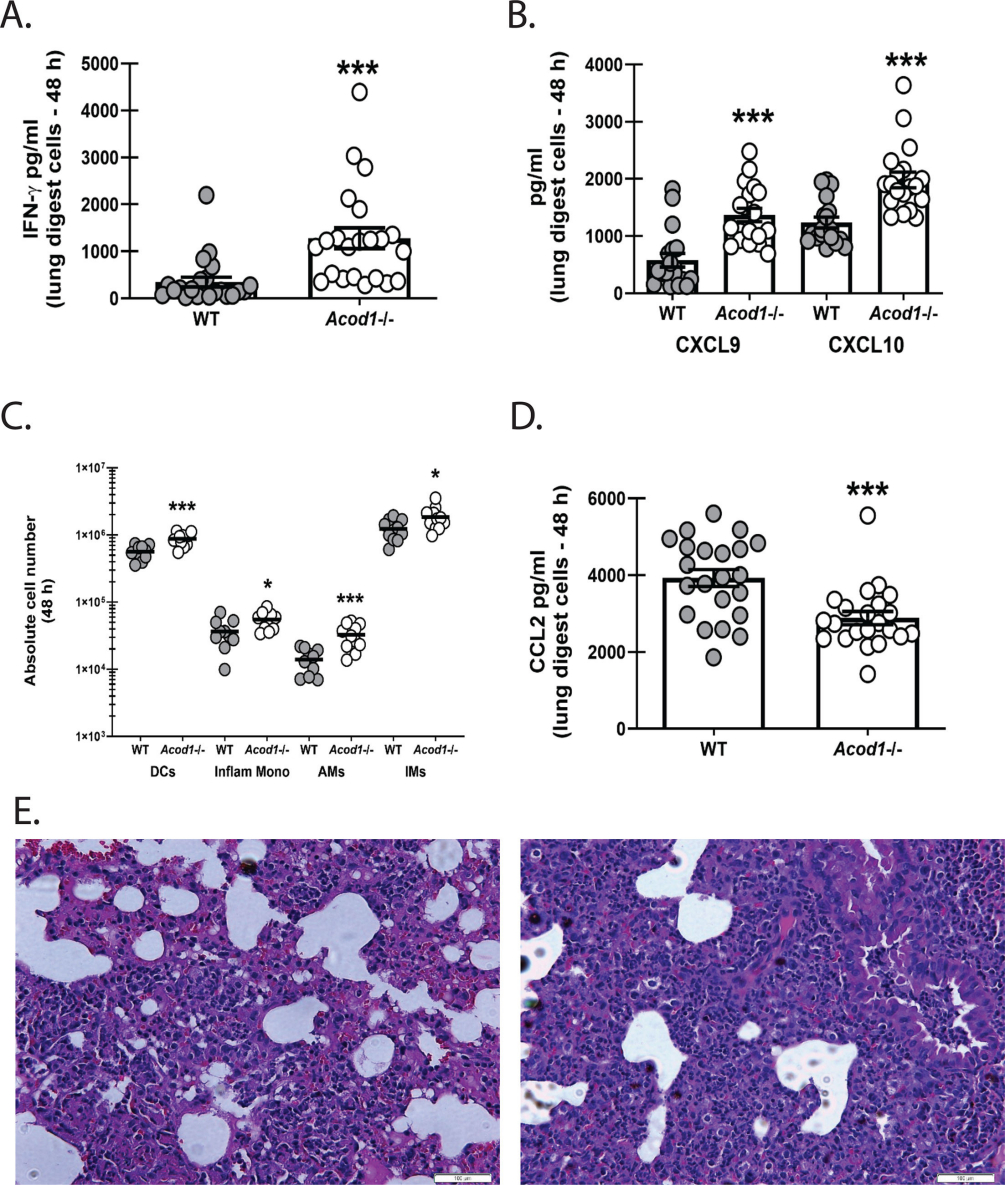
The absence of itaconate results in increased type 1 responses during *Aspergillus fumigatus* lung infection. C57BL/6NJ wild-type (WT) and itaconate-deficient (*Acod1*−/−) mice were challenged intratracheally with 7 × 10^7^ *A. fumigatus* conidia. At 48 h post-challenge, lungs were collected, enzymatically digested and cultured in duplicate for 24 h. (**A**) IFN-γ and (**B**) CXCL9 and CXCL10 levels were quantified in co-culture supernatants by Luminex-based Milliplex assessment. The panels illustrate cumulative data from eight independent studies (*n* = 5 or 6 mice per group, per time point, per study). (**C**) Lung digest cells were isolated as in panel **A**. Dendritic cells (DCs), inflammatory monocytes, alveolar macrophages (AMs), and interstitial macrophages (IMs) were quantified by flow cytometry. The panels illustrate cumulative data from two independent studies (*n* = 4 or 5 mice per group, per time point, per study). (**D**) CCL2 levels were quantified in co-culture supernatants by Luminex-based Milliplex assessment. The panel illustrates cumulative data from eight independent studies (*n* = 5 or 6 mice per group, per time point, per study). For all graphs, each symbol represents an individual mouse, the line within a given group represents the mean, and *, **, and *** represent a *P* value of <0.05, <0.01, and <0.001, respectively (unpaired two-tailed Student’s *t*-test). (**E**) Representative H&E stained lung sections from WT mice (left) and *Acod1*−/− mice (right). Original magnification 40×. Scale bar equals 100 μm.

IFN-γ is a well-studied cytokine in *A. fumigatus* host defense, with studies cumulatively showing that IFN-γ activates PMNs to kill *A. fumigatus* hyphae (34–36) as well as positively affects monocyte and macrophage responses (37, 38). Results show that in addition to elevated IL-17A and IL-22 production, *Acod1*−/− mice also demonstrated increased levels of IFN-γ (Fig. 3A) as well as the IFN-γ-associated chemokines CXCL9 and CXCL10 (Fig. 3B). CXCL9 and CXCL10 are involved in the recruitment of multiple cell types, including dendritic cells and monocytes (39). To this end, both of these cell types were increased in the lungs of *Acod1*−/− mice, along with higher numbers of alveolar macrophages and interstitial macrophages (Fig. 3C). Curiously, CCL2, which is a recognized mediator of monocyte and monocyte-derived DC recruitment during *A. fumigatus* infection (40), was significantly lower in *Acod1*−/− mice (Fig. 3D). H&E staining of lung tissue 48 h after *A. fumigatus* exposure also demonstrated evidence of higher inflammatory cell recruitment (Fig. 3E). These data suggest that itaconate also functions in the regulation of type 1-mediated immune responses during fungal pneumonia.

### Itaconate negatively regulates innate antifungal effector activity against *A. fumigatus*

**Fig 4:**
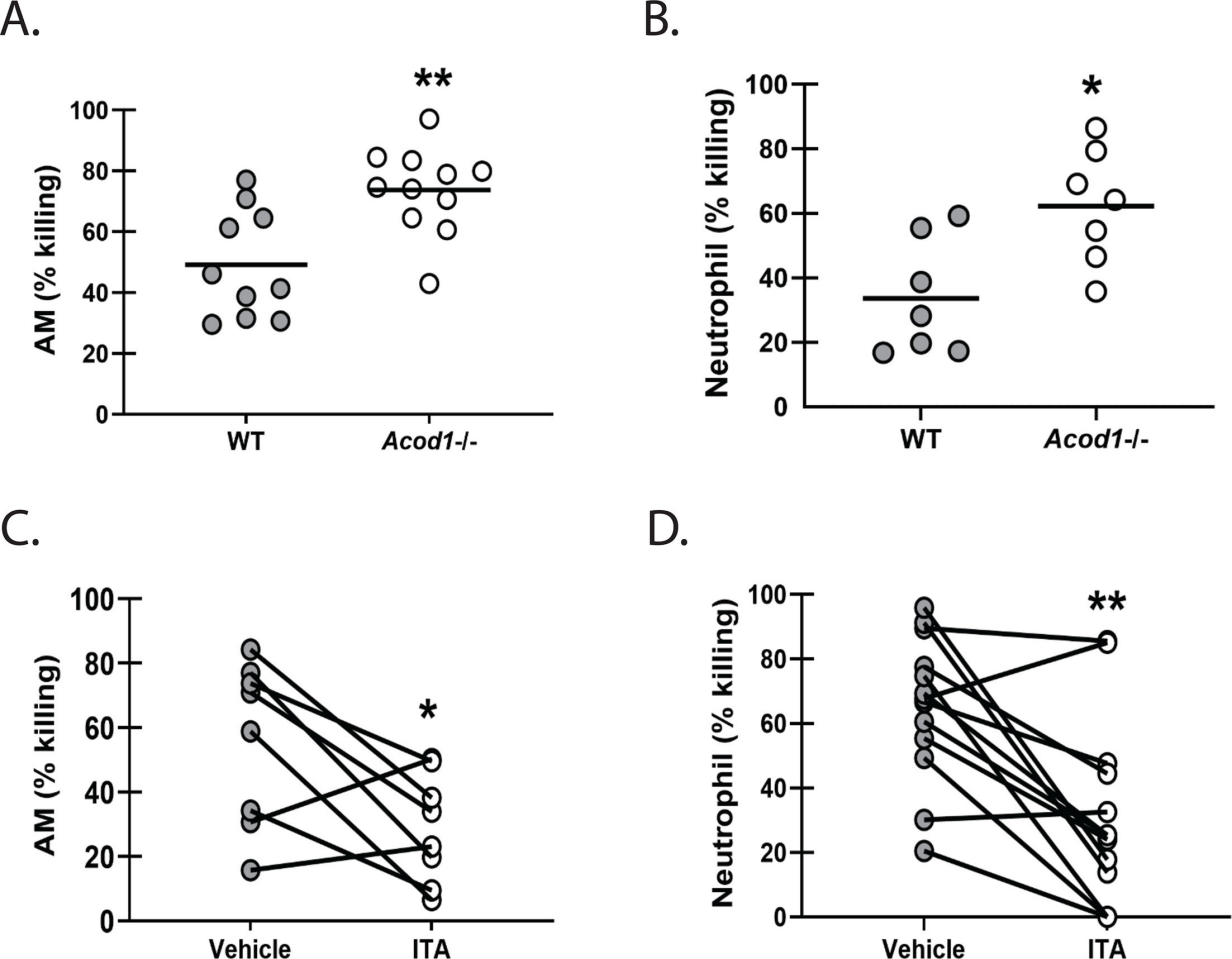
Itaconate negatively regulates innate antifungal effector activity against *A. fumigatus*. (**A**) Alveolar macrophages (AMs) were isolated via bronchoalveolar lavage from naïve C57BL/6NJ wild-type (WT) and itaconate-deficient (*Acod1*−/−) mice and plated 1:1 with *A. fumigatus* resting conidia for 24 h. Total contents of each well were collected, and fungal killing was assessed by real-time PCR analysis of *A. fumigatus* 18S rRNA levels. Data are expressed as a percentage of fungi killed. The panel illustrates cumulative data from three to four independent studies (*n* = 2 or 3 mice per group per study). Each symbol represents an individual mouse, the line within a given group represents the mean, and ** represents a *P* value of <0.01 (unpaired two-tailed Student’s *t*-test). (**B**) Bone-marrow neutrophils from naïve WT and *Acod1*−/− mice were isolated using a Neutrophil Isolation Kit (Cat. #130-097-658, Miltenyi Biotec) per manufacturer’s instructions and cultured 1:1 with *A. fumigatus* swollen conidia. Total contents of each well were collected, and fungal killing was assessed by real-time PCR analysis of *A. fumigatus* 18S rRNA levels. Data are expressed as percentage of fungi killed. The panel illustrates cumulative data from two independent studies (*n* = 3 or 4 mice per group per study). Each symbol represents an individual mouse, the line within a given group represents the mean, and ** represents a *P* value of <0.01 (unpaired two-tailed Student’s *t*-test). (**C**) AMs from C57BL/6NJ wild-type (WT) mice were cultured as in panel **A** in the presence or absence of itaconate (10 mM) for 24 h. Total contents of each well were collected, and fungal killing was assessed by real-time PCR analysis of *A. fumigatus* 18S rRNA levels. Data are expressed as percentage of fungi killed. The panel illustrates cumulative data from two independent studies (*n* = 4 mice per group per study). Each symbol represents an individual mouse cultured in the presence of vehicle or itaconate and * represents a *P* value of < 0.05 (paired two-tailed Student’s *t*-test). (**D**) Neutrophils were isolated as in panel **B** and cultured in the presence or absence of itaconate (10 mM) for 24 h. Total contents of each well were collected, and fungal killing was assessed by real-time PCR analysis of *A. fumigatus* 18S rRNA levels. Data are expressed as percentage of fungi killed. The panel illustrates cumulative data from three independent studies (*n* = 3–5 mice per group per study). Each symbol represents an individual mouse cultured in the presence of vehicle or itaconate, and * represents a *P* value of <0.05 (paired two-tailed Student’s *t*-test).

Alveolar macrophages and neutrophils are early effector cells in the lung during fungal pneumonia (41, 42). We hypothesize that in the absence of itaconate, the fungicidal capacity of these innate effector cells may be modulated. Data in Fig. 4A indicate that alveolar macrophages isolated from the lungs of naïve *Acod1*−/− mice displayed enhanced killing of *A. fumigatus* compared to those isolated from naïve WT BL/6NJ mice. Results also showed that neutrophils isolated from the bone marrow of naïve *Acod1*−/− mice had twofold higher fungal killing capacity than neutrophils from WT mice (Fig. 4B). Conversely, adding exogenous itaconate to alveolar macrophages (Fig. 4C) or neutrophils (Fig. 4D) from naïve WT BL/6NJ mice lowered their ability to kill *A. fumigatus*. These data suggest that in the absence of itaconate, innate effector cells such as alveolar macrophages and neutrophils have intrinsically higher antifungal activity. In contrast, the addition of itaconate inhibited the ability of alveolar macrophages and neutrophils to effectively kill *A. fumigatus*.

### Itaconate negatively regulates reactive oxygen species production by alveolar macrophages, but not neutrophils

**Fig 5:**
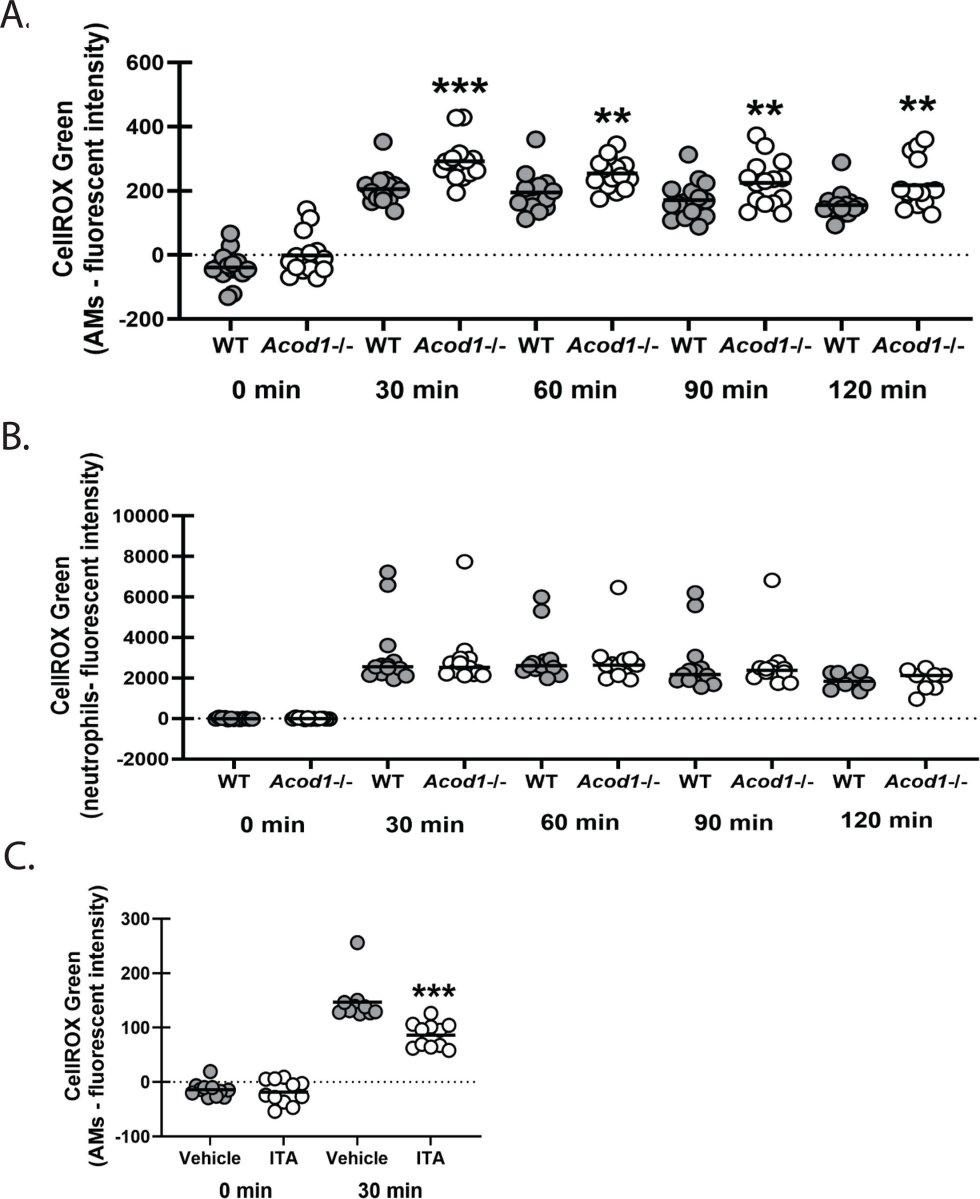
Itaconate negatively regulates reactive oxygen species production by alveolar macrophages. (**A**) Alveolar macrophages (AMs) were isolated via bronchoalveolar lavage and (**B**) neutrophils isolated from bone marrow from C57BL/6NJ wild-type (WT) and itaconate-deficient (*Acod1*−/−) mice and plated 1:1 with *A. fumigatus* resting (AMs) or swollen (neutrophils) conidia in the presence of CellROX Green (5 µM). Reactive oxygen species levels were measured using a fluorescent plate reader (485 nm) every 30 min for 2 h. The panels illustrate cumulative data from three independent studies (*n* = 3 or 4 mice per group per study). Each symbol represents an individual mouse, and the line within a given group represents the mean, and ** and *** represent a *P* value of <0.01 and <0.001, respectively (unpaired two-tailed Student’s *t*-test). (**C**) Alveolar macrophages (AMs) were isolated as in panel **A** and plated 1:1 with *A. fumigatus* resting (AMs) conidia in presence or absence of itaconate (10 mM) and in the presence of CellROX Green (5 µM). Reactive oxygen species levels were measured using a fluorescent plate reader (485 nm) for 30 min. The panel illustrates cumulative data from three independent studies (*n* = 3 or 4 mice per group per study). Each symbol represents an individual mouse, the line within a given group represents the mean, and *** represents a *P* value of <0.001(unpaired two-tailed Student’s *t*-test).

Data above demonstrated that alveolar macrophages and neutrophils from naive *Acod1*−/− mice had intrinsically higher antifungal activity. As the generation of reactive oxygen species (ROS) is a major antifungal killing mechanism of these cells (43), we questioned whether the absence of itaconate resulted in increased ROS production. Results in Fig. 5A show that alveolar macrophages from naive *Acod1*−/− mice demonstrated heightened ROS production at multiple time points after stimulation with *A. fumigatus* conidia compared to those from WT BL/6NJ mice. Surprisingly, neutrophils from naive *Acod1*−/− mice stimulated with *A. fumigatus* did not show an increase in ROS production (Fig. 5B). Culturing AMs in the presence of exogenous itaconate resulted in lower ROS induction (Fig. 5C). These data suggest that itaconate regulates an unknown pathway of ROS induction in alveolar macrophages, whereas itaconate-mediated regulation of neutrophil antifungal activity is independent of ROS.

### Itaconate deficiency in macrophages and dendritic cells, but not neutrophils, results in enhanced lung clearance of *Aspergillus fumigatus*

**Fig 6:**
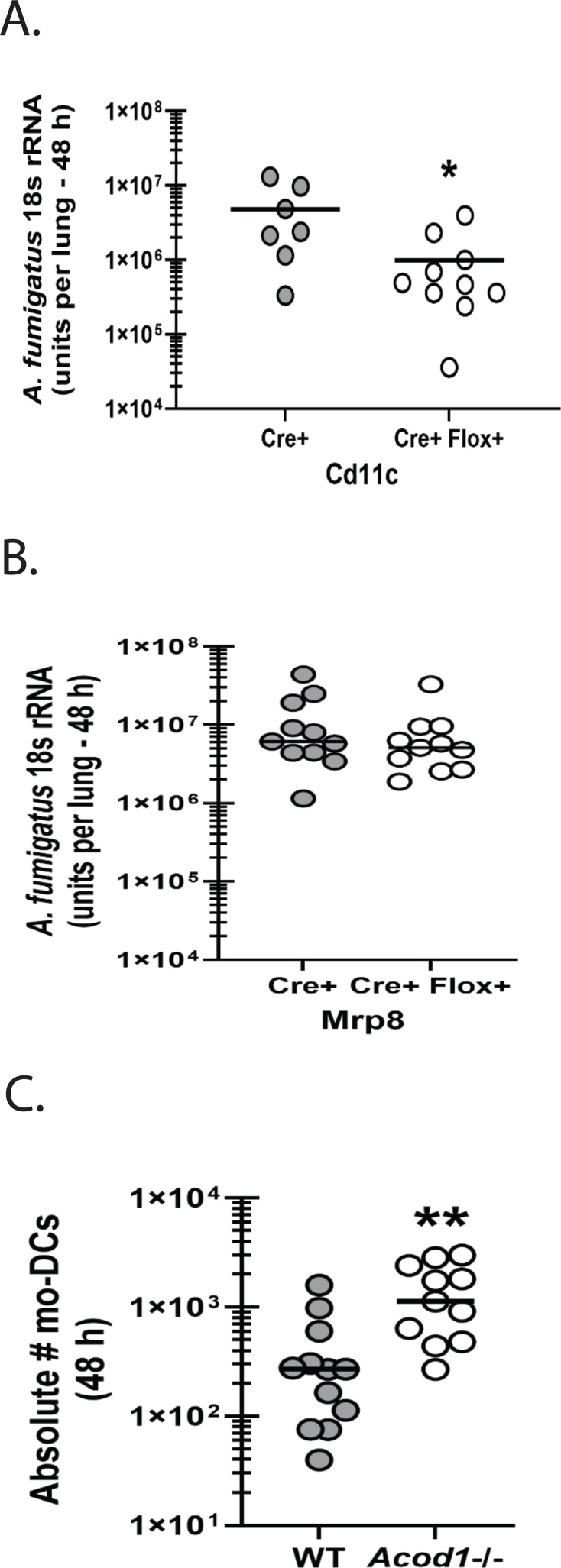
Itaconate deficiency in macrophages and dendritic cells, but not neutrophils, results in enhanced lung clearance of *Aspergillus fumigatus*. (**A**) Cd11c Cre+ (control) and Cd11c Cre+/Acod1*^flox/flox^* were challenged intratracheally with 7 × 10^7^ *A. fumigatus* conidia. Lung fungal burden at 48 h post-challenge was assessed by real-time PCR analysis of *A. fumigatus* 18S rRNA levels. The panel illustrates cumulative data from two independent studies (*n* = 3–5 mice per group per study). Each symbol represents an individual mouse, the line within a given group represents the mean, and * represents a *P* value of <0.05 (unpaired two-tailed Student’s *t*-test). (**B**) Mrp8 Cre+ (control) and Mrp8 Cre+/Acod1*^flox/flox^* were challenged intratracheally with 7 × 10^7^ *A. fumigatus* conidia. Lung fungal burden at 48 h post-challenge was assessed by real-time PCR analysis of *A. fumigatus* 18S rRNA levels. The panel illustrates cumulative data from two independent studies (*n* = 5 or 6 mice per group per study). Each symbol represents an individual mouse, and the line within a given group represents the mean. (**C**) WT and *Acod1*−/− mice were challenged intratracheally with 7 × 10^7^ *A. fumigatus* conidia. At 48 h post-challenge, lungs were collected and enzymatically digested and mo-DCs were quantified by flow cytometry. The panel illustrates cumulative data from two independent studies (*n* = 5 or 6 mice per group per study). Each symbol represents an individual mouse, the line within a given group represents the mean, and ** represents a *P* value of <0.01 (unpaired two-tailed Student’s *t*-test).

Our data thus far show that in the absence of itaconate, alveolar macrophages and neutrophils have enhanced antifungal activity against *A. fumigatus,* whereas exogenous itaconate lowers the capacity of alveolar macrophages and neutrophils to kill *A. fumigatus*. We, therefore, questioned whether specific deletion of itaconate (*Acod1*) in CD11c+ cells had an impact on *A. fumigatus* lung clearance. For this, we crossed Cd11c Cre mice with Acod1 flox/flox mice, which deletes *Acod1* expression in both lung macrophages and DCs, and exposed them to an acute challenge with *A. fumigatus*. Data in Fig. 6A indicate that itaconate deficiency in macrophages recapitulated the level of fungal clearance observed in germline *Acod1*-deficient mice (Fig. 1D). In contrast, specific deletion of itaconate in neutrophils via crossing Mrp8 Cre mice with Acod1 flox/flox mice resulted in no difference in lung fungal burden at 48 h (Fig. 6B). Previous studies have shown that monocyte-derived DCs (mo-DCs) are important effector cells against *A. fumigatus* (44). As data in Fig. 6A indicated that *Acod1* deficiency specifically in CD11c+ cells resulted in augmented *A. fumigatus* clearance and that CD11c is a primary marker for mo-DCs (45), we questioned whether there were differences in this DC subset in the absence of itaconate. Data in Fig. 6C show that *Acod1*−/− mice have a significant increase in the numbers of mo-DCs in the lung after *A. fumigatus* exposure. Thus, the absence of itaconate in CD11c+ cells (macrophages and DCs) is sufficient for improving resistance to *A. fumigatus* lung infection.

### Itaconate deficiency protects against fungus-induced mortality during corticosteroid-mediated immunosuppression

**Fig 7:**
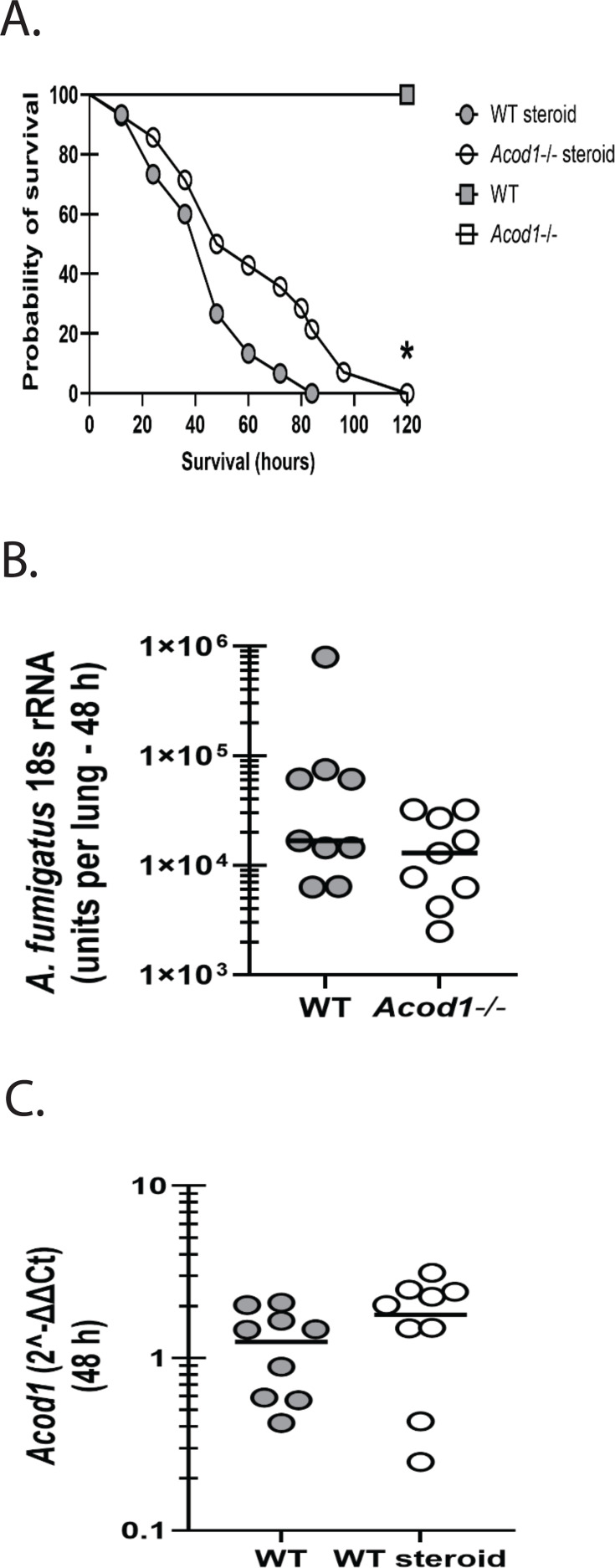
Itaconate deficiency protects against fungus-induced mortality during corticosteroid-mediated immunosuppression. (**A**) WT and *Acod1*−/− mice were left untreated or administrated triamcinolone acetonide (Kenalog, 40 mg/kg intraperitoneally) for 24 h. Thereafter, mice were lightly anesthetized with isoflurane and administered 7 × 10^6^ *A. fumigatus* conidia in a volume of 50 μL intratracheally and survival followed for 4 days. The panel illustrates cumulative data from three independent studies (*n* = 5 or 6 mice per group per study). The graph shows the probability of survival and * represents a *P* value of <0.05 (log rank [Mantel-Cox] test). (**B**) Lung fungal burden at 48 h post-challenge was assessed by real-time PCR analysis of *A. fumigatus* 18S rRNA levels. The panel illustrates cumulative data from two independent studies (*n* = 4 or 5 mice per group per study). Each symbol represents an individual mouse, and the line within a given group represents the mean. (**C**) *Acod1* gene expression was quantified in whole lung from (**B**) by real-time PCR and normalized to HPRT. Data are expressed as 2−ΔΔCt. The panel illustrates cumulative data from two independent studies (*n* = 4 or 5 mice per time point per study).

Corticosteroid treatment is the primary risk factor for developing invasive pulmonary aspergillosis (IPA) (3). A recent study reported that the anti-inflammatory properties of corticosteroids involve reprogramming of macrophage mitochondrial metabolism, resulting in increased and sustained production of itaconate and consequently, inhibition of the inflammatory response (46). To this end, we questioned whether the absence of itaconate could modulate susceptibility to invasive fungal infection associated with corticosteroid-mediated immunosuppression. Results in Fig. 7A show that *Acod1*−/− treated with steroids had a small, but significant, survival advantage when exposed to *A. fumigatus*. However, when fungal burden was assessed at the time point when mortality was first occurred (48 h), there was no difference between WT and *Acod1*−/− mice (Fig. 7B). *Acod1* mRNA levels in whole lung trended higher in WT mice treated with steroids (48 h) (Fig. 7C). Thus, itaconate promotes susceptibility to corticosteroid-associated invasive fungal infection.

### Exogenous itaconate modulates human monocyte-derived macrophage antifungal activity

**Fig 8:**
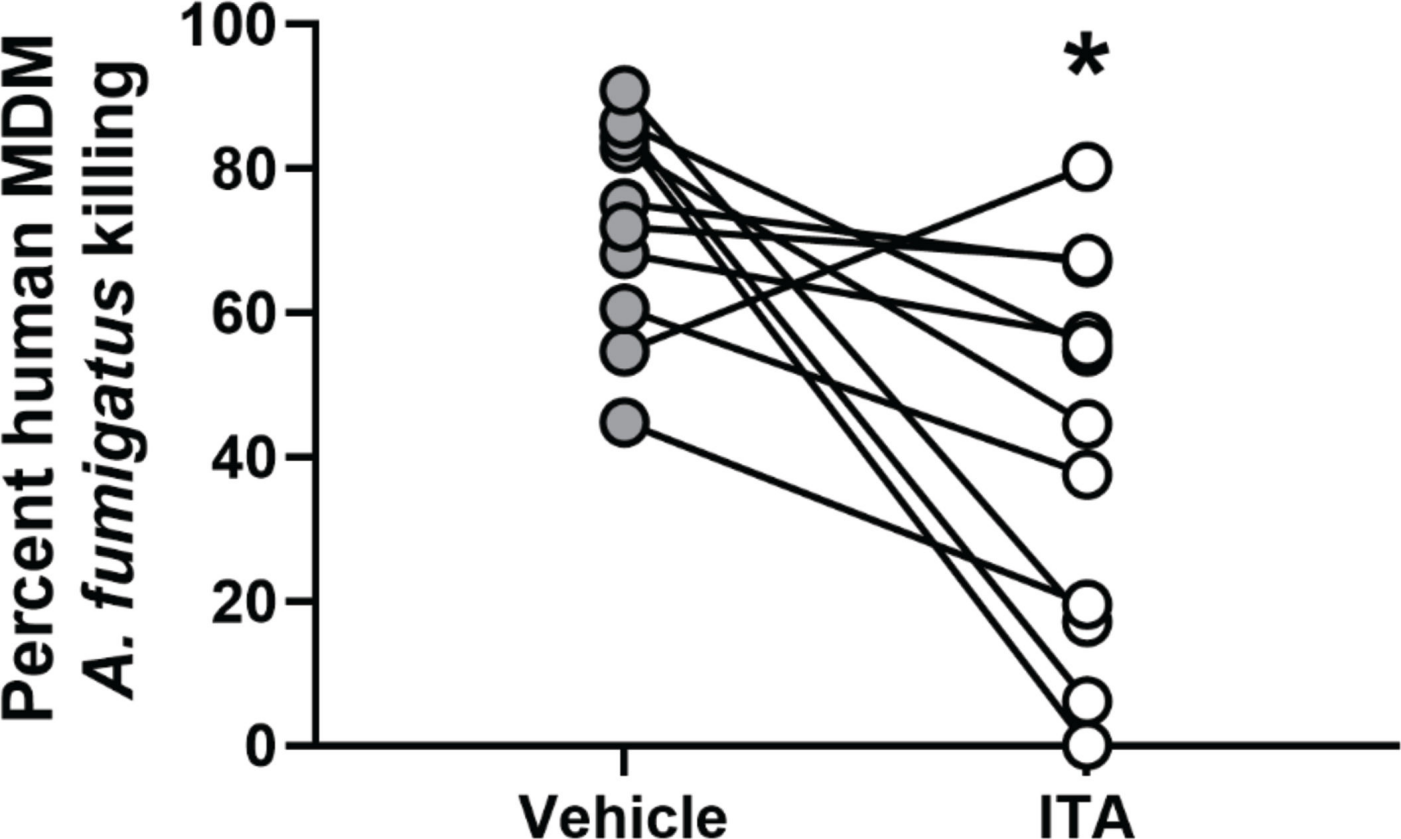
Exogenous itaconate modulates human monocyte-derived macrophage antifungal activity. Peripheral blood samples from healthy human volunteers were drawn into BD Vacutainer Blood Collection Tubes and peripheral blood mononuclear cells isolated via Ficoll-Paque PLUS. Monocytes were isolated using a human Pan Monocyte Isolation Kit and cultured with human recombinant M-CSF and ImmunoCult-SF Macrophage Medium for 4 days. Monocyte-derived macrophages (MDMs) (1 × 10^5^) were co-cultured at a 1:1 effector-to-target ratio with live *A. fumigatus* resting conidia in the presence of vehicle or itaconate (10 mM) for 24 h. Total contents of each well were collected, and fungal killing was assessed by real-time PCR analysis of *A. fumigatus* 18S rRNA levels. Data are expressed as a percentage of fungi killed. The figure illustrates cumulative data from 10 individual donors assessed in 3 independent experiments. Each symbol represents MDMs generated from an individual donor cultured in the presence of vehicle or itaconate, and * represents a *P* value of < 0.05 (paired two-tailed Student’s *t*-test).

Monocyte-derived macrophages are extensively employed to assess human responses to *A. fumigatus* (47–49). To increase the significance of our findings, we question whether itaconate could modulate the antifungal activity of human innate immune cells. For this, we isolated whole blood from healthy human volunteers and generated monocyte-derived macrophages (MDMs). MDMs were cultured with *A. fumigatus* resting conidia in the presence of exogenous itaconate for 24 h followed by assessment of fungal killing. Results in Fig. 8 show that similar to murine alveolar macrophages, itaconate negatively affects the ability of human MDMs to kill *A. fumigatus*. These data indicate that the inhibitory effects of itaconate on the antifungal capacity of macrophages are not specific to a single species.

## DISCUSSION

The absence of ACOD1/IRG1 results in increased susceptibility to *Mycobacterium tuberculosis* as a result of excessive lung inflammation, which included elevated recruitment of neutrophils in the presence of increased production of IL-1β, IL-6, IL-17A, IL-12p70, and IFN-γ (17). *Acod1*−/− (*Irg1*−/−) mice have also been shown to be susceptible to *Klebsiella pneumoniae* lung infection, demonstrating a 2–3 log increase in lung bacterial burden in the presence of increases in IL-6, CXCL1, CXCL2, and CCL3 (15). Outside of the lung, the absence of itaconate results in increased susceptibility to Zika virus (50). In contrast, the absence of itaconate during lung infection with *Staphylococcus aureus* (51) results in better pathogen clearance, but surprisingly in the presence of lower levels of G-CSF, CXCL1, and CXCL2 and no differences in inflammatory cell numbers (51). Similar results were observed for *Pseudomonas aeruginosa* (52) and *Mycoplasma pneumonia* (53). Itaconate itself has been shown to have antimicrobial effects against SARS-CoV2 (54) and *Salmonella typhimurium* (55). Itaconate does not appear to influence susceptibility to the viral pathogen influenza (17) or systemic infection with *Listeria monocytogenes* (17). Here, we interrogated the role of itaconate in regulating the immune response to the highly inflammatory opportunistic mold *Aspergillus fumigatus*.

Initial observations demonstrated that the absence of itaconate resulted in a 2.5-fold reduction in lung fungal burden at 48 h after challenge with *A. fumigatus*. Unlike many bacterial and viral infections, log/multi-log differences in fungal burden are not required in order to see dramatic differences in survival or immunological readouts. To this end, we have reported similar reductions in lung fungal burden in mice deficient in the receptor for IL-33 (*Il1rl1*−/−) (20) and mice deficient in acidic mammalian chitinase (*Chia*−/−) (56). It is interesting to note that like *Acod1*−/− mice in the current report, both *Il1rl1*−/− and *Chia*−/− mice demonstrated an enhancement in type 17 responses (IL-17A and IL-22 production) (20, 56). An additional similarity in these studies is the presence of significantly higher IL-1β in all three strains. In our IL1RL1 study, we reported that IL-1β and PGE2 levels correlated with increased type 17 responses in *Il1rl1*−/− mice. We showed that both PGE2 and type 17 responses were significantly reduced in the absence of IL-1 receptor signaling, whereas inhibiting PGE2 in *Il1rl1*−/− mice via the COX-2 inhibitor celecoxib or with an inhibitor of microsomal prostaglandin E synthase-1 abrogated type 17 responses (20). Collectively, our previous results indicate a central role for an IL-1β/PGE2 axis in driving type 17 responses during *A. fumigatus* lung infection. Likewise, the elevated type 17 responses we observed in *Chia*−/− mice also occurred in the presence of elevated IL-1β and PGE2 (56). Our data now implicate itaconate as an additional factor that regulates type 17 responses via IL-1β and PGE2, as lung cells from *Acod1*−/− produced significantly more IL-1β and PGE2 than those from WT mice. We have further reported that iNKT cells are required for early (24 h) production of the type 17 cytokine IL-22, whereas γδ T cells are required for IL-22 production at 48 h after *A. fumigatus* exposure (19). Regarding the cells responsible for IL-17A and IL-22 production in the absence of itaconate, our data primarily support a role for γδ T cells, as these cells were elevated in *Acod1*−/− mice at 48 h, whereas there were no differences in iNKT cells or neutrophils. One potential caveat of our study is that the *A. fumigatus* strain we employ is readily recognized via the beta-glucan receptor Dectin-1. Therefore, we cannot exclude the possibility that our findings with itaconate may be more applicable to those strains in which beta-glucan primarily drives responses such as type 17 responses.

Despite some level of investigative depth regarding the role of IFN-γ in *A. fumigatus* lung infection, the precise mechanisms of how IFN-γ promotes antifungal immunity is limited. IFN-γ treatment in experimental models has been shown to augment protection against infection (57, 58) although the induction and function of IFN-γ is negatively affected by corticosteroid or cyclophosphamide-mediated immune suppression (59, 60). As referenced earlier, *in vitro* studies have shown that IFN-γ can augment human neutrophil-mediated hyphal damage (34, 35), presumably via ROS induction (36). In contrast, the effects of IFN-γ on monocytes and macrophages are mixed, with studies reporting positive (37, 38) or no (59, 61) effect. Likewise, both alveolar and interstitial macrophage levels were increased in *Acod1*−/− mice, both of which may be responding to the elevated levels of IFN-γ observed in these mice leading to increased antifungal activity. Indeed, culturing alveolar macrophages from *Acod1*−/− in the presence of IFN-γ-augmented alveolar macrophage killing (Fig. S1). Finally, elevated IFN-γ levels in *Acod1*−/− mice correlated with the levels of CXCL9 and CXCL10. Polymorphisms in *Cxcl10* are associated with increased risk of developing invasive aspergillosis (62), whereas mice deficient in CXCR3, the receptor for CXCL10, have increased susceptibility to *A. fumigatus* lung infection (44). Susceptibility in the latter is thought to be due to defective recruitment of neutrophil-activating plasmacytoid DCs (44), which are required for *A. fumigatus* elimination (63). Although we did not observe any differences in pDCs in the absence of itaconate (data not shown), despite lower levels of CCL2, inflammatory monocytes were elevated in *Acod1*−/− mice. Inflammatory monocytes have been shown to produce CXCL9 and CXCL10 during *A. fumigatus* lung infection (44), as well as respond to IFN-γ, leading to their differentiation into monocyte-derived DCs (64). Collectively, although the itaconate-mediated axis regulating inflammatory monocyte/monocyte-derived DC recruitment is unclear, augmented *A. fumigatus* clearance in the absence of itaconate could also be attributed to increased presence of these cell types.

An intriguing finding from our study is the observation that in the absence of itaconate, alveolar macrophages and neutrophils had enhanced killing of *A. fumigatus in vitro*. This did not appear to be an artifact of *Acod1*−/− deficiency, as the addition of itaconate to alveolar macrophages and neutrophils from normal mice impaired their ability to kill *A. fumigatus*. Moreover, we verified these observations by showing that itaconate impairs the antifungal killing capacity of human monocyte-derived macrophages. The impact of itaconate on macrophage responses was further reinforced by showing that *Acod1* deletion in CD11c+ macrophages resulted in augmented fungal clearance. However, *Acod1* deletion in neutrophils had no effect on fungal burden, further highlighting a predominant role for itaconate in regulating macrophage antifungal responses. We also observed an increase in monocyte-derived DCs (mo-DCs) in *Acod1*−/− mice, which have been shown to play a role in *A. fumigatus* elimination (44). Therefore, the possibility exists that mo-DCs are also responsible for the phenotype observed in mice with CD11c-specific *Acod1* deletion. Moreover, an additional immunoregulatory function of itaconate may be to influence the survival, recruitment, or development of mo-DCs. The generation of reactive oxygen species in innate immune cells is a required element of optimal host defense against *A. fumigatus* (65). This is accentuated by the observation that individuals with chronic granulomatous disease, who have mutations in one of the five subunits of the nicotinamide adenine dinucleotide phosphate (NADPH) oxidase enzyme complex, are exquisitely susceptible to infection with *A. fumigatus* as a result of impaired ROS generation (66). Our data revealed that itaconate regulated ROS production in alveolar macrophages, but surprisingly not neutrophils. This is presumably via negative regulation of succinate dehydrogenase (67). In addition, 4-octyl itaconate has been shown to inhibit mitochondrial ROS induction in response to LPS in the MH-S alveolar macrophage cell line (68).

A previous study reported that corticosteroids amplify itaconate production during inflammation, which subsequently suppresses the inflammatory response (46). This study reported that corticosteroids were not effective at lowering inflammation in *Acod1*−/− mice subjected to a model of lung injury with LPS exposure, an experimental arthritis model, or an allergic asthma model (46). A compelling finding in our study was the observation that itaconate-deficient mice treated with corticosteroids had increased survival after exposure to *A. fumigatus*. Although we did not observe a difference in fungal burden at the time mortality commenced in the corticosteroid model, we cannot exclude the possibility that fungal burden may be different at other time points. Moreover, the difference in survival between corticosteroid-treated WT vs *Acod1*−/− mice may be a result of differences in pulmonary function. Nevertheless, as corticosteroid therapy is the dominant risk factor for developing IPA (3–6), our data demonstrating that itaconate restricts innate immune responses to *A. fumigatus* now establish a potential link behind corticosteroid-mediated susceptibility to *A. fumigatus* lung infection.

## SUPPLEMENTAL MATERIAL

Figure S1 mbio.01491-26-s0001.pdf Effects of IFN-γ and IL-17A on AM antifungal activity against *A. fumigatus*.

ASM does not own the copyrights to Supplemental Material that may be linked to, or accessed through, an article. The authors have granted ASM a non-exclusive, world-wide license to publish the Supplemental Material files. Please contact the corresponding author directly for reuse.
