# Supplementary material for: Itaconate negatively regulates innate immunity during fungal pneumonia
Source: mBio. 2026 Jul 27;17(8):e01491-26. doi: 10.1128/mbio.01491-26 (PMC13463890; doi:10.1128/mbio.01491-26)
Supplement: Supplementary file 1 [file mbio.01491-26-s0001.pdf]

Supplemental Figure 1

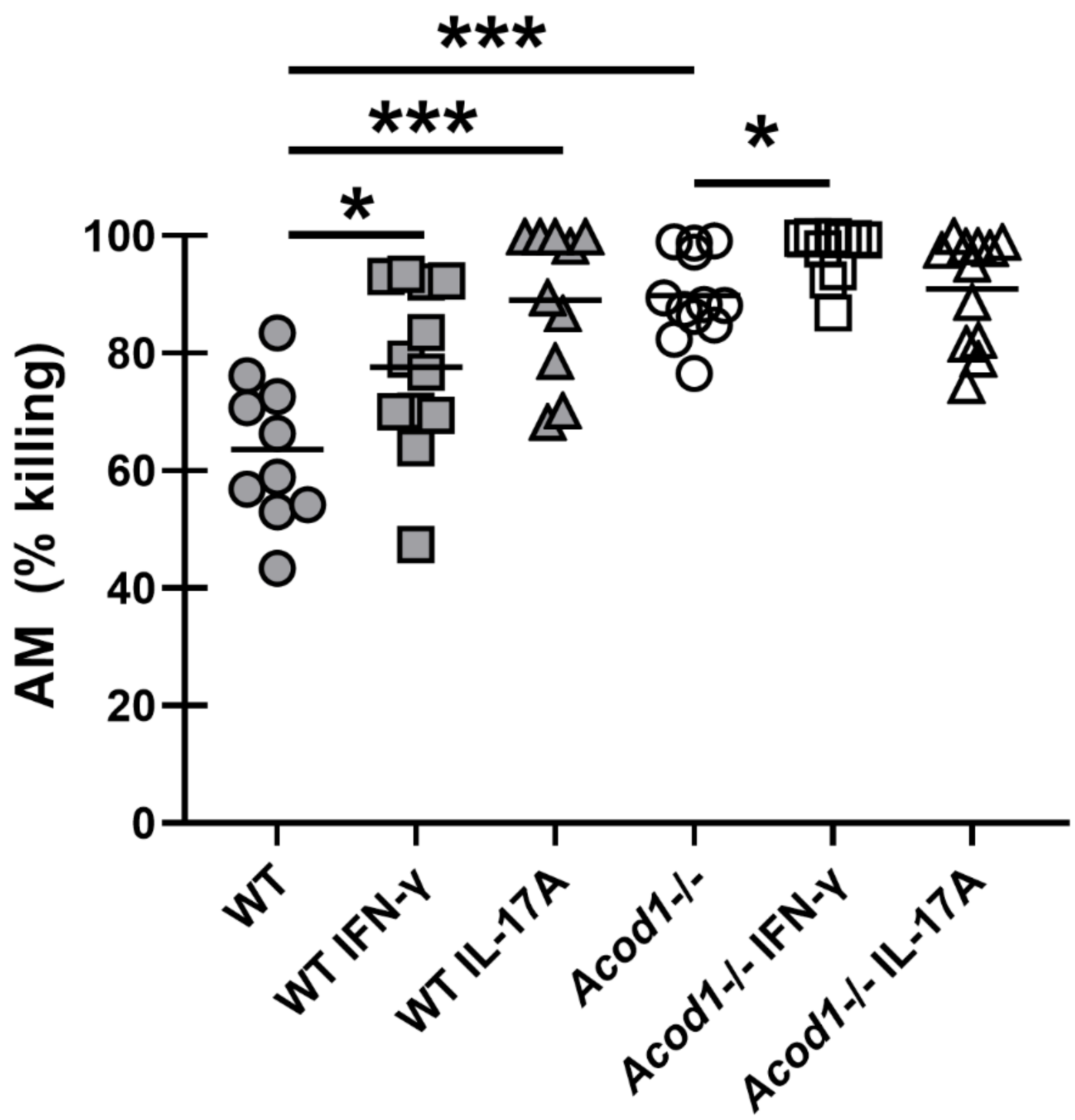

Supplemental Figure 1. The effects of IFN-γ and IL-17A on AM antifungal activity against *A. fumigatus*. Alveolar macrophages (AMs) from naïve WT vs. *Acod1*-/- mice were isolated via bronchoalveolar lavage and cultured 1:1 with *A. fumigatus* resting conidia in the presence of IFN-γ or IL-17A (10 ng/ml). *A. fumigatus* conidia cultured with vehicle or each cytokine served as controls for viability. After 24 h, the total contents of each well were collected, and fungal killing was assessed by real-time PCR analysis of *A. fumigatus* 18S rRNA levels. Data are expressed as mean percentage of fungi killed. Each symbol represents an individual mouse and the line represents the mean. The Figures illustrate cumulative data from 2-3 individual experiments. * and *** represents a P value of <0.05 and 0.001 (two-tailed Student's t test).
